# The effect of Citrus aurantium inhalation aromatherapy on chemotherapy-induced nausea and vomiting in breast cancer patients: a randomized controlled trial

**DOI:** 10.1186/s12906-025-05052-0

**Published:** 2025-10-07

**Authors:** Parsa Tabei, Zahra Molazem, Mozhgan Rivaz, Parvin Ghaem Maghami, Niloofar Ahmadloo

**Affiliations:** 1 Department of Nursing, School of Nursing and Midwifery, Student Research Committee, Shiraz University of Medical Sciences, Shiraz, Iran; 2 Department of Nursing, School of Nursing and Midwifery, Shiraz University of Medical Sciences, Shiraz, Iran; 3 Associate Professor of Nursing Education, School of Nursing and Midwifery, Shiraz University of Medical Sciences, Shiraz, Iran; 4 Department of Radio–oncology, School of medicine, Breast Disease Research Center, Namazi Teaching Hospital, Shiraz University of Medical Sciences, Shiraz, Iran

**Keywords:** Aromatherapy, Chemotherapy-induced nausea and vomiting, Breast cancer, Chemotherapy, Citrus aurantium

## Abstract

**Background:**

Breast cancer (BC) is the second most common cancer globally and the most prevalent among women. Chemotherapy, a primary treatment, frequently induces distressing side effects like nausea and vomiting. This study evaluated the efficacy of Citrus aurantium inhalation aromatherapy in reducing chemotherapy-induced nausea and vomiting (CINV) in women with BC.

**Methods:**

In this CONSORT-compliant parallel-group randomized controlled trial (registered at IRCT: 20240305061177N1), 92 BC patients at Motahari Clinic were randomly allocated (between April to August 2024) to an aromatherapy group (*n* = 46) or a control group (*n* = 46). Alongside standard antiemetic medications, the aromatherapy group inhaled two drops of C. aurantium essential oil applied to the philtrum, while the control group received sunflower oil. The Rhodes Index assessed nausea, vomiting, and retching over five days.

**Results:**

Significant reductions in mean total scores for nausea, vomiting, and retching were observed in the aromatherapy group compared to the control group from days 3 to 5 (*P* < 0.05), with no significant differences on days 1 and 2. Total symptom scores significantly decreased from Day 1 to Day 5 in the aromatherapy group while the control group showed no change.

**Conclusion:**

C. aurantium aromatherapy significantly alleviated CINV during the delayed phase (days 3–5). Given its safety, affordability, and accessibility, this intervention offers a promising complementary approach for managing CINV in BC patients.

**Trial registration:**

This trial was registered with the Iranian Registry of Clinical Trials (IRCT20240305061177N1, march 17, 2024).

**Supplementary Information:**

The online version contains supplementary material available at 10.1186/s12906-025-05052-0.

## Background

Breast cancer (BC) is the second most common cancer worldwide [1] and the most common cancer among women [2]. Approximately 10% of women aged 13–90 are affected by this disease. Although BC primarily affects women, it is also diagnosed in men, accounting for less than 1% of cases [3]. Common treatments for BC include surgery, chemotherapy, radiation therapy, and immunotherapy, which may be used in combination [4]. Chemotherapy plays a critical role in managing BC; however, it is associated with numerous complications, such as nausea and vomiting [5, 6]. Nausea and vomiting are among the most common and distressing complications of chemotherapy [7]. Despite the development of antiemetic drugs, chemotherapy-induced nausea and vomiting (CINV) remains a serious issue for many patients, potentially disrupting their cancer treatment [8, 9]. Various guidelines recommend the use of 5HT3 receptor antagonists, dexamethasone, and neurokinin antagonists in patients receiving highly emetic chemotherapeutic (HEC) drugs such as Adriamycin and Doxorubicin [10, 11]. Nevertheless, approximately 60% of patients still experience nausea, and 30% suffer from vomiting, even with these antiemetics [12]. These medications can also lead to side effects, including heartburn, insomnia, headaches, dizziness, constipation, diarrhea, and dry mouth [13]. As a result, researchers are exploring less invasive, safer, and more cost-effective approaches to manage CINV.

Aromatherapy, a branch of complementary and alternative medicine (CAM), provides a complementary treatment method. Aromatherapy refers to the use of essential oils extracted from the aromatic parts of plants to treat or relieve physical and emotional symptoms; these oils are more cost-effective and less invasive than chemical drugs [14]. Few studies have investigated the effects of herbal medicines on CINV [15].

Breast cancer patients did not experience relief from chemotherapy-induced nausea and vomiting (CINV) after being treated with ginger aromatherapy [16]. On the other hand, the application of aromatherapy with peppermint oil has resulted in significant antiemetic effects in breast cancer patients undergoing chemotherapy [17]. These findings, coupled with the ongoing CINV challenge warrant deeper exploration into more effective management strategies considering the existing antiemetic therapies do not provide a sufficient solution.

Bitter orange (Citrus aurantium) may be a new Phyto therapeutic agent, with fascinating bioactive elements being identified including limonene, linalool, and β-myrcene [18]. Some history of its use in aromatherapy is not enough to justify its efficacy for treating CINV. However, preclinical evidence supports more research in the field. Limonene, one of the constituents of C. aurantium essential oil, has been proven to affect serotonin and dopamine pathways in the central nervous system, which are central to nausea and vomiting, especially CINV [19, 20].

A 2018 preclinical study demonstrated limonene’s anti-inflammatory, antioxidant, and gastroprotective effects in rodent models, supporting its relevance to CINV [21]. Likewise, linalool exhibits neuroprotective and anti-inflammatory properties in animal studies [22], potentially reducing gastrointestinal inflammation—a significant contributor to CINV [23]. D-limonene, a primary component of Citrus aurantium essential oil, has demonstrated incredible gastroprotective and anti-inflammatory characteristics upon preclinical trial. These processes may potentially contribute to the mitigation of inflammation, whilst maintaining the gastrointestinal system, for D-limonene to assist with alleviating the chemotherapy-induced nausea [24, 25].

Additionally, C. aurantium suppressed pro-inflammatory cytokines (e.g., TNF-α, IL-1β) in murine models, suggesting an anti-inflammatory mechanism relevant to CINV [26]. Inhalation of C. aurantium essential oil stimulates the olfactory system, which has direct connections to the limbic system and the vomiting center in the brainstemو thereby potentially modulating nausea and vomiting responses [14], offering a neuromodulatory pathway to mitigate symptoms. Furthermore, a systematic review indicates that C. aurantium may reduce stress and anxiety in preclinical and clinical settings [27], and reducing these psychological factors may indirectly alleviate CINV [28].

There have been publications on consensus guidelines across the globe within multiple organizations (e.g. ASCO, NCCN, MASCC/ESMO) that have developed coordination strategies for CINV with recommendations for use of triple drug antiemetic regimens with 5HT3 receptor antagonists, neurokinin-1 (NK1) receptor antagonists, and dexamethasone in patients—therefore integrated with emetic chemotherapy (HEC) [29]. Despite some progress, there remain substantial gaps as the appropriate management of delayed-phase CINV continues to challenge researchers and clinicians, reinforcing the need for aromatherapy in addition to pharmacological therapies.

Considering this preclinical evidence, we hypothesized that inhalation aromatherapy with C. aurantium would decrease the intensity of chemotherapy-induced nausea and vomiting (CINV) in breast cancer patients receiving AC-T chemotherapy. C. aurantium inhalation aromatherapy would add a new, evidence-informed therapy as a complementary treatment with the standard antiemetic treatment. Therefore, this study aimed to answer the question: Does Citrus aurantium inhalation aromatherapy reduce chemotherapy-induced nausea and vomiting (CINV) in women with breast cancer?

## Methods

Trial Design: This study was a parallel, double-blinded, randomized controlled trial and implemented at the Motahari Center affiliated with Shiraz University of Medical Sciences. The article was written in accordance with the CONSORT 2010 statement.

Inclusion and Exclusion Criteria: The recruitment criteria were women between the ages of 18 and 80 who had a confirmed medical diagnosis of breast cancer stage 2 or 3 with no evidence of distant metastasis in their medical files, who were treated with the same standard AC-T chemotherapy protocol with the same dose, and who experienced nausea and vomiting of any severity during or after chemotherapy. There were at least two remaining chemotherapy courses with the same protocol: not pregnant; literate; having a healthy sense of smell and taste; not suffering from mental and emotional diseases; not having allergies to plants, bitter orange; not suffering from diseases such as asthma, allergic diseases, and pulmonary disease; not using other complementary medicine methods during the study; not having epilepsy; and using the same antiemetics. Exclusion criteria included unwillingness to continue participating in the study, failure to use the aroma according to the provided plan, concurrent chemotherapy with radiotherapy, and suffering from any disease or condition that leads to nausea and vomiting (e.g., liver or kidney failure, digestive problems, acute stages of hepatitis B, gastrointestinal obstruction, and brain malignancies).

The participants were not allowed to have previously used aromatherapy or complementary medicine approaches to treat their nausea and vomiting because it was important to have a uniform baseline from which to evaluate the influence of the intervention. To ensure homogeneity, all participants were aromatherapy-naïve, meaning they had never used aromatherapy for any purpose prior to the study.

Sample Size: To determine a sample size, a Type I error (alpha) of 0.05 and a Type II error (beta) of 0.20 were considered, along with a 95% confidence level and 80% power. The required sample size was calculated using G*Power software, referencing previous studies that indicated the effect size of 0.59 [13, 30]. This analysis revealed that a sample size of 47 participants would be needed for both the intervention and control groups. To account for a potential 6.5% attrition rate, the sample size was increased to 50 participants in each group, resulting in a total of 100 participants.

$$\:\mathbf{n}=\varvec{\uprho\:}\frac{{\left({\mathbf{z}}_{1-\frac{\varvec{\upalpha\:}}{2}}+{\mathbf{z}}_{1-\varvec{\upbeta\:}}\right)}^{2}}{{\left(\mathbf{d}\right)}^{2}}$$

$$\:\mathbf{d}=\frac{({{\mathbf{S}}_{1}}^{2}+{{\mathbf{S}}_{2}}^{2})}{{({\varvec{\upmu\:}}_{2}-{\varvec{\upmu\:}}_{1})}^{2}}$$

Randomization: Participants were randomly assigned to either the intervention group or control group using a permuted block randomization method that included 25 blocks of 4. The random allocation sequence was generated by the research team using Random Allocation software.

**Fig. 1:**
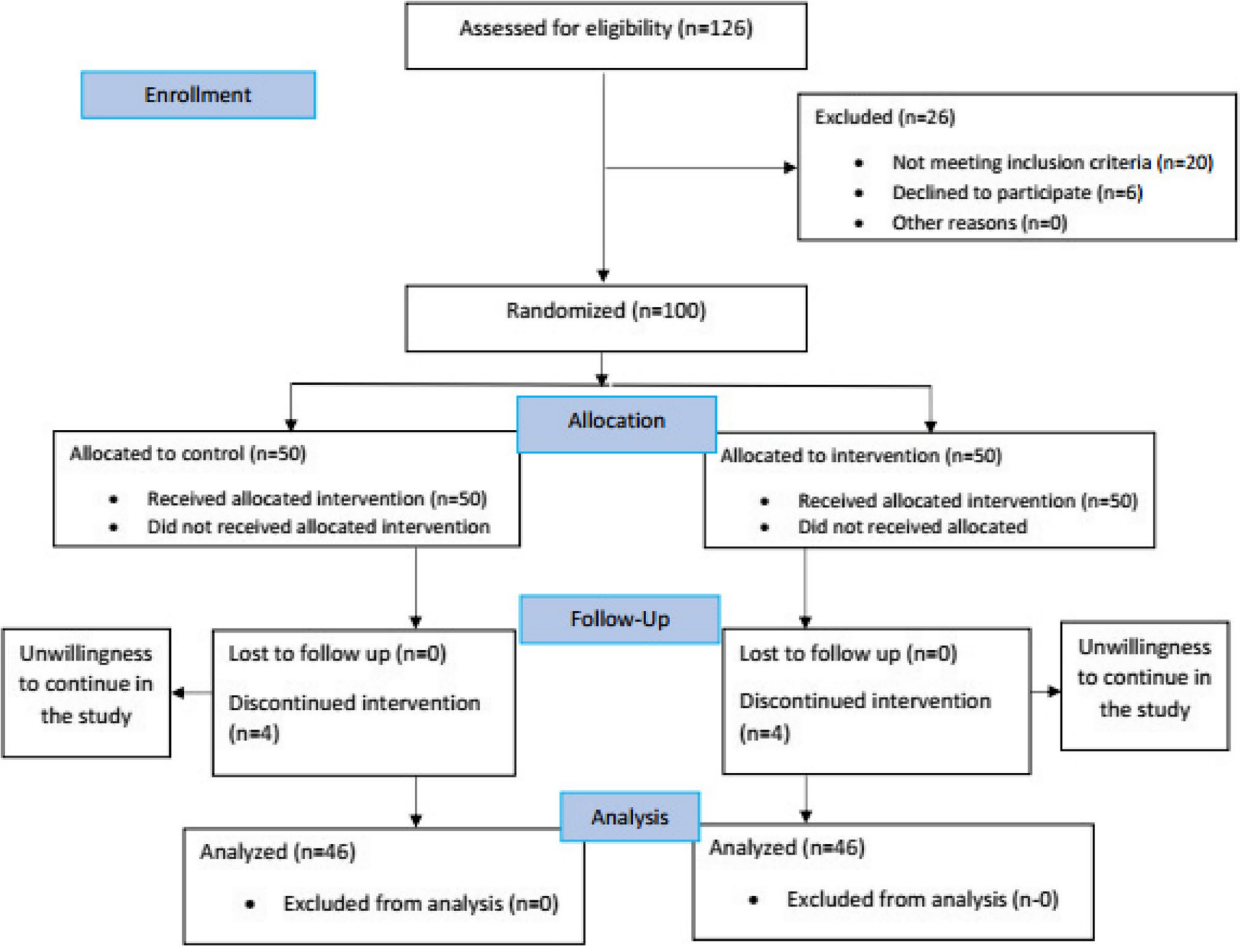
CONSORT flow diagram

Interventions: This double-blinded, single-center, randomized controlled trial was conducted from August 2023 to December 2024. During the intervention, 8 participants dropped out of the study due to choosing not to proceed their treatment. A total of 92 BC patients referred to the Motahari Clinic were randomly assigned to two groups: the control group (46 patients) and the aromatherapy group (46 patients) (Fig. 1).

All participants provided written informed consent before enrolling in the study. This occurred during the patient’s first appointment at the chemotherapy clinic after the eligibility screening. Every participant was undergoing AC-T chemotherapy, a standard treatment regimen consisting of Adriamycin (A) and Cyclophosphamide (C) followed by Taxanes (T). This is a frequent treatment approach for patients with early-stage breast cancer, and received identical premedication, which included dexamethasone (8 mg IV), ondansetron (4 mg IV), and chlorpheniramine (10 mg IV). The participants in the aromatherapy group received bitter orange oil, whereas those in the control group received sunflower oil (selected as an odorless placebo with no reported antiemetic effects to ensure blinding integrity) in identical unlabeled bottles supplied oils by “Tabib Daru” Co. A literature search confirmed no prior reports of sunflower oil’s use for CINV. The patients did not know the nature of the oil and whether they were in the intervention or control group, nor the provided baseline questionnaires mentioned the oil type.

They were all outpatients having undergone active chemotherapy. Hence the intervention was conducted in the home of the patient, not the hospital. This enabled data collection in a controlled setting but with the patient’s own environmental context. There was no chance of cross-contamination of stimulant and no left-over residual smells from either group. Thereby allowing research validity because there was no chance of the smell of the intervention group influencing the control group.

The participants were given chances several times to follow the instructions in the use of the intervention.

(Aromatherapy and Placebo) and analyzed with the Rhodes questionnaire on the first visit to the chemotherapy clinic after giving informed consent, and before starting the intervention.

Participants were trained in the process of applying the intervention by the research team in a live demonstration, and then they were given written materials given by the research team explaining the procedure for applying a two-drop ardoroma oil on the philtrum (the area between the upper lip and nose). They were asked to breathe normally for 20 min, and this procedure was repeated three times daily at 09:00, 15:00, and 21:00 for five days. The dosage and timing were based on prior aromatherapy studies demonstrating efficacy within this timeframe [31]. Both groups completed the Rhodes index daily for five days.

The choice of five days for the intervention period was based on previous studies that demonstrated the delayed phase of CINV typically occurs between 24 h and up to 5 days post-chemotherapy [28]. This timeframe aligns with the pharmacokinetic profile of many chemotherapeutic agents and their impact on the gastrointestinal tract [32].

### Measurement tool

#### Rhodes index of nausea, vomiting, and retching

The Rhodes index, developed by Rhodes and McDaniel in 1999, was used to assess nausea, vomiting and retching [28]. The purpose of this questionnaire is to evaluate both subjective and objective factors related to nausea, vomiting, and retching in various situations [32]. The original version of this questionnaire has demonstrated good validity, with a correlation coefficient of 0.87, and strong reliability, with a Cronbach’s alpha coefficient of 0.89–0.97 [33]. This index employs a numerical rating scale (0–10) for intensity, with higher scores indicating greater symptom severity. It also measures the severity (none, mild, moderate, great, severe) and frequency (none, 1–2, 3–4, 5–6, ≥ 7 episodes) of nausea, vomiting, and retching. Additionally, the index assesses the amount of vomiting (none, small (≤½ cup), moderate (½–2 cups), large (2–3 cups), very large (> 3 cups)) and the duration of nausea (none, ≤ 1 h, 2–3 h, 4–6 h, > 6 h) [4]. The index assesses symptoms experienced in the preceding 24 h. Scores are categorized as follows: 0–8 as mild, 9–16 as moderate, 17–24 as severe, and 25–35 as very severe [28]. The Persian version of this index is approved by Cronbach’s alpha = 0.87 [34].

### Blinding

To ensure proper blinding, the statistician who analyzed the data and the patients were unaware of the group assignments. Additionally, the clinicians and nurses at the Motahari Center were also blinded to the groups.

### Statistical methods

All the analyses were done on SPSS version 23. Quantitative data is reported using mean and standard deviation while qualitative data is reported using frequencies and percentages. Chi-square test was used to determine the relationship between categorical demographic variables (e.g. marital status, educational level, family history of breast cancer, smoking, alcohol consumption, and presence of underlying diseases) and group allocation (intervention and control) as shown in Table 1.

**Table 1:** Distribution of demographic and clinical characteristics

Variable	Aromatherapy Group (*n* = 46)	Control Group (*n* = 46)	Total (*n* = 92)	*p*-value*
Marital Status				0.29
- Single	7 (15.2%)	11 (23.9%)	18 (19.6%)	
- Married	39 (84.8%)	35 (76.1%)	74 (80.4%)	
Education Level				0.61
- Below Diploma	19 (41.3%)	16 (34.8%)	35 (38.0%)	
- High School Diploma	18 (39.1%)	24 (52.2%)	42 (45.7%)	
- Bachelor’s Degree	7 (15.2%)	5 (10.9%)	12 (13.0%)	
- Master’s/Other	2 (4.3%)	1 (2.2%)	3 (3.3%)	
Family History of BC				0.62
- Yes	10 (21.7%)	12 (26.1%)	22 (23.9%)	
- No	36 (78.3%)	34 (73.9%)	70 (76.1%)	
Smoking History				1.00
- Yes	0 (0.0%)	1 (2.2%)	1 (1.1%)	
- No	46 (100.0%)	45 (97.8%)	91 (98.9%)	
Alcohol Consumption				1.00
- Yes	0 (0.0%)	1 (2.2%)	1 (1.1%)	
- No	46 (100.0%)	45 (97.8%)	91 (98.9%)	
Underlying Disease				0.27
- Yes	13 (28.3%)	18 (39.1%)	31 (33.7%)	
- No	33 (71.7%)	28 (60.9%)	61 (66.3%)	
Age (Mean ± SD)	49.43 ± 9.9	46.91 ± 10.11	48.17 ± 10.07	0.23

*BC* Breast Cancer *SD* Standard Deviation

*chi-square test was done

An independent samples t test was used to assess the relationships between quantitative variables by comparing means across two levels of a variable and between two groups. Given that the sample size was over 30, parametric tests were applied, following the central limit theorem to ensure robust results.

### Ethical considerations

This research was conducted in accordance with the ethical principles of the Declaration of Helsinki and gained approval from the Shiraz University of Medical Sciences (Approval Code: IR.SUMS.NUMIMG.REC.1403.003) Ethics Committee. The trial is registered in the Iranian Registry of Clinical Trials Registration Number: IRCT20240305061177N1 and registered on March 17, 2024. All participants gave written informed consent before the study began.

Participants received information related to the purpose of the investigation, data collection process, some of the possible harms, and the right to withdraw from the investigation at any time with no impact on your medical care at any time. Personal data was stored confidentially to protect the privacy and anonymity of participants in this research study.

## Results

In this study, 92 eligible participants were randomly assigned to the control or aromatherapy group. The demographic characteristics, including an overall mean age of 48 ± 10.07 years, were similar between the two groups (all *p* > 0.05, Table 1).

The majority of the participants were married (80.4%), held a high school diploma (45.7%), reported no family history of BC (76.1%), and did not smoke or consume alcohol (98.9%). Additionally, 66.3% of the participants indicated that they had no specific underlying disease.

Independent samples t tests were conducted to compare the mean scores of vomiting, nausea, belching, and total scores between the groups (Tables 2, 3, 4 and 5).

**Table 2:** Comparison of vomiting scores between groups over time

Day	Aromatherapy Group (Mean ± SD)	Control Group (Mean ± SD)	*p*-value*
Day 1	0.69 ± 1.75	1.11 ± 1.90	0.28
Day 2	0.66 ± 1.65	0.85 ± 1.77	0.58
Day 3	0.50 ± 1.32	1.18 ± 1.93	0.05
Day 4	0.37 ± 0.72	1 ± 1.97	0.04
Day 5	0.29 ± 0.59	0.87 ± 1.52	0.01

*SD* standard deviation

*Independent-samples t-test was done

**Table 3:** Comparison of nausea scores between groups over time

Day	Aromatherapy Group (Mean ± SD)	Control Group (Mean ± SD)	*p*-value*
Day 1	4.18 ± 3.11	5.09 ± 3.73	0.20
Day 2	4.70 ± 3.22	5.33 ± 3.98	0.40
Day 3	4.20 ± 3.40	6.22 ± 4.20	0.01
Day 4	3.53 ± 3.23	4.90 ± 3.68	0.06
Day 5	2.50 ± 3.21	4.61 ± 3.60	0.004

*SD* standard deviation

*Independent-samples t-test was done

**Table 4:** Comparison of retching scores between groups over time

Day	Aromatherapy Group (Mean ± SD)	Control Group (Mean ± SD)	*p*-value*
Day 1	1.42 ± 1.87	1.83 ± 1.89	0.29
Day 2	1.40 ± 2.20	2.24 ± 2.41	0.07
Day 3	1.59 ± 2.20	2.83 ± 2.84	0.02
Day 4	1.16 ± 1.73	1.94 ± 2.32	0.06
Day 5	0.77 ± 1.41	1.61 ± 2.02	0.02

*SD* standard deviation

*Independent-samples t-test was done

**Table 5:** Comparison of total symptom scores between groups over time

Day	Aromatherapy Group (Mean ± SD)	Control Group (Mean ± SD)	*p*-value*
Day 1	6.29 ± 5.80	8.03 ± 5.71	0.15
Day 2	6.74 ± 5.99	8.42 ± 6.73	0.20
Day 3	6.29 ± 5.87	10.22 ± 7.29	0.005
Day 4	5.05 ± 4.77	7.83 ± 6.51	0.02
Day 5	3.55 ± 4.56	7.09 ± 5.91	0.002

*SD* standard deviation

*Independent-samples t-test was done

The aromatherapy group consistently exhibited lower mean vomiting scores compared to the control group across all five days. While differences on Days 1 and 2 were not statistically significant, significant reductions were observed in the aromatherapy group on Days 3 (*p* = 0.05), 4 (*p* = 0.04), and 5 (*p* = 0.01).

The aromatherapy group demonstrated a steady decrease in nausea scores over the five days, while the control groups scores remained relatively higher throughout the study. Significant differences were found in favor of the aromatherapy group on Days 3 (*p* = 0.01) and 5 (*p* = 0.004).

The aromatherapy group demonstrated a steady decline in retching scores from Day 1 to Day 5. Significant group differences were documented on Day 3 (*p* = 0.02) and Day 5 (*p* = 0.02), which indicates that aromatherapy was effective in reducing retching.

The overall symptom scores representing the symptoms of nausea, vomiting, and retching decreased significantly for the aromatherapy group over the five days. Significant group differences were seen on Day 3 (*p* = 0.005), Day 4 (*p* = 0.02), and Day 5 (*p* = 0.002), all favoring the aromatherapy group. Comparison of within-group Symptom Scores Day 1 and Day 5 are depicted in Table 6.

**Table 6:** Within-Group comparison of symptom scores day 1 and day 5 in the aromatherapy and control groups

Symptom	Aromatherapy Group			Control Group		
	t-statistic	p-value*	ES**	t-statistic	p-value*	ES**
Vomiting	12.47	< 0.001	0.97	0.97	0.337	0.05
Nausea	5.41	< 0.001	0.28	0.28	0.778	0.03
Retching	3.50	0.001	1.1	1.10	0.279	0.07
Total	5.14	< 0.001	1.62	1.62	0.111	0.10

*Paired t-test was used for within-group comparisons

**Effect Size

The aromatherapy group reported a statistically significant decrease in their vomit scores from Day 1 to Day 5 (t = 12.47, *p* < 0.001) demonstrating a large effect size (Cohen’s d = 0.97). The control group did not have a significant change (*p* = 0.337). Nausea scores significantly decreased in the aromatherapy group (t = 5.41, *p* < 0.001) with a small effect size (Cohen’s d = 0.28). The control group did not exhibit a significant change from Day 1 to Day 5 in nausea scores (*p* = 0.778). The aromatherapy group did have a significant decrease in retching scores (t = 3.50, *p* = 0.001), demonstrating a large effect size (Cohen’s d = 1.10), while the control group had no significant change (*p* = 0.279). The total symptoms scores decreased significantly in the aromatherapy group (t = 5.14, *p* < 0.001), demonstrating a very large effect size (Cohen’s d = 1.62) and no significant change in the control group (*p* = 0.111).

The data indicate that aromatherapy significantly reduces nausea, vomiting, and retching symptoms over time, with substantial effect sizes, particularly in vomiting and total symptom scores. In contrast, the control group did not exhibit significant improvements, underscoring the potential efficacy of aromatherapy as a complementary treatment for these symptoms.

### Harms

Potential harms included negative reaction to C. aurantium, discomfort due to application, potential unblinding, and potential foe delayed/missed standard CINV treatment. No adverse effects were noted during the course of the research period. Neither group of participants noted any discomfort, allergic reactions or complications related to the use of essential oils or placebo.

## Discussion

This study revealed a significant decrease in the mean vomiting score over time in the aromatherapy group, indicating that C. aurantium aromatherapy effectively reduces chemotherapy-induced vomiting. This finding aligns with previous research on the use of aromatherapy for managing CINV. For example, Alikamali (2022) reported that C. aurantium aromatherapy reduced vomiting [15]. Additionally, studies using other scents have reported similar benefits; Rambod et al. (2023) reported reduced vomiting scores with lemon aromatherapy in Iranian postoperative patients [35], and Istiroha (2023) reported similar results with peppermint aromatherapy in Indonesian women with BC [36].

It should be noted that no statistically significant differences were observed for Days 1 or 2 for either group (*p* > 0.05), and therefore it is not possible to assert that C. aurantium aroma therapy showed a therapeutic effect within this acute time period of chemotherapy-induced nausea and vomiting.

Considering the clinical relevance of changes are often subjective due to no established MCID for the Rhodes Index, there were 43.6% relative reduction in the intervention group and 11.7% reduction in the control group, and improvements in patient- reported outcomes, suggest clinically meaningful effects are possible. In addition, in the aromatherapy group, the within-group analysis demonstrated vomiting, nausea, retching, total symptom score showed large to very large effect sizes (Cohen’s d comparing day 1 to 5 = 0.97 to 1.62). Overall these data support the rationale for clinical practice of Citrus aurantium aromatherapy; the potential impact appears to exceed a statistical measure, with possible clinical implications to support practice. Most importantly, effect sizes for all symptoms were much larger in the aroma therapy group relative to the control group, which clearly suggests clinically important change due to the intervention.

The results corroborate others that physician perspectives on 30–50% improvement parameters is clinically important [35, 36]. Future research to explore a cancer-specific Rhodes MCID will require a clear commitment to strategies engaging patients, secondly regarding longitudinal follow-up surveys, and thirdly the use of anchor-based scaling.

Discrepancies with studies on ginger where no significant effects on CINV [5] may be due to the types of aromas used, intervention time frames, or differences with the patient population and chemotherapy regimen used. The lack of efficacy in ginger studies might stem from differences in bioactive compounds (e.g., gingerols vs. limonene) or receptor specificity. For instance, limonene in C. aurantium directly modulates serotonin pathways, whereas ginger’s mechanisms may target prostaglandin inhibition, which is less relevant to acute CINV.

Importantly, the lack of significant symptom relief among the control group during the same time frame reinforces the specificity of the intervention effect and lessens the potential for the improvements to be attributed to spontaneous recovery or placebo. The multiple contributing factors could explain the reason why the therapeutic effects of “Citrus aurantium” aromatherapy were delayed until the improvement was noted from Day 3 and afterward. Firstly, delay phase CINV or the acute phase of chemotherapy induced nausea and vomiting is noted within the first 24 h after chemotherapy treatment and is primarily driven by activation of serotonin (5HT3) receptors. Routine antiemetic therapy, which includes 5HT3 antagonists and corticosteroids, usually works well during this phase. With this, aromatherapy might not have any augmenting effects during the first two days [37].

Ultimately, the beneficial effects of aromatherapy are seen in tandem with any observed clinical improvement in nausea and vomiting which is particularly evidenced in the delayed phase of chemotherapy-induced nausea and vomiting (CINV) that represents the time period 24 to 120 h post-treatment concerning alternative emetogenic pathways including activation of substance P and neurokinin-1 (NK1) receptors. Maybe during this later phase is when the aromatherapy treatment works better because there may be less pharmacological coverage with more non-pharmacological methods available [38].

Repeated exposure and accumulation of particular bioactive compounds in the C. aurantium essential oil, especially D-limonene, may explain the delayed therapeutic effects; therefore, reinforcing the hypothesis. In vitro D-limonene’s anti-inflammatory, gastroprotective, and serotonin-modulating activity has been demonstrated; however, they may need multiple dosing regimens to obtain their full therapeutic benefits [24, 25]. The Rhodes Index total scores together with this incremental and cumulative symptom reduction in contrast to the anticipated time frame provides further evidence to the hypothesis that sustained exposure to fragrance relieves symptoms associated with delayed CINV.

Supportive care patients may be more likely to benefit from slowly escalating psychological and physiological effects of aromatherapy intervention. Additional research is required to determine these factors and refine aromatherapy application and dosing strategies However, these mechanisms may take longer to modulate nausea and vomiting pathways, particularly in the delayed phase when other contributing factors—such as inflammation and psychological stress—become more prominent [25].

Unlike ginger, which primarily exhibits anti-inflammatory properties, limonene’s dual action on serotonin receptors and gastroprotection may explain its delayed-phase efficacy. This mechanistic distinction underscores the importance of aroma selection based on targeted CINV phases.

To address this gap, future studies should consider earlier initiation of aromatherapy (e.g., before chemotherapy administration) or combine it with standard antiemetics in the acute phase. Additionally, further research is needed to explore the synergistic effects of Citrus aurantium with pharmacological agents targeting both acute and delayed phases of CINV.

The divergent nausea trends between groups suggest that C. aurantium may mitigate chemotherapy-induced nausea through sustained neuromodulatory effects, contrasting with the transient symptom relief seen in the control group. This aligns with Zorba et al. [39], where multi-scent blends enhanced efficacy, but conflicts with ginger studies [5], possibly due to aroma-receptor interaction variability.

Furthermore, the retching scores of the aromatherapy group decreased significantly over time, whereas those of the control group initially increased until day 3, followed by a slight decrease. These findings suggest that aromatherapy may also be effective in reducing retching. While previous studies have often focused on the severity and duration of nausea and vomiting, our study uniquely examined all three symptoms: nausea, vomiting, and retching.

The swift reduction of symptoms in the intervention group supports the hypothesis of C. aurantium being not just an adjunct, but a true therapeutic use, while the slower reduction of symptoms in the control group is likely attributable to a physiological habituation. Across days 3 and 5 the total mean Rhodes Index scores were statistically significantly different among the cerebral side groups and the control group. The mean; C. aurantium scores followed an overlapping pattern with the anticipated expectation that C. aurantium exposure would have accumulative/sequential advantages in mean colony score, although the benefits were not reflected in the applications of treatment.

The delayed retrieval of the conventional clinical outcomes aligns with the delayed pharmacodynamic timing that is inherently characteristic of complementary therapy, particularly a distance form of complementary therapy, consistent with aromatherapy which requires uninterrupted exposure to obtain a clinically measurable outcome. Future trials should focus on delayed CINV measurement that address the notion that effects may extend beyond the time of the aromatherapy or treatment exposure when patients returned for delayed measures. These findings are supported by those of Sulistyarini et al. (2022), who reported that three days of ginger aromatherapy effectively reduced nausea, vomiting, and retching [40]. In conclusion, this study provides evidence that C. aurantium aromatherapy can be an effective complementary therapy for managing CINV. While our findings support C. aurantium’s role in CINV management, generalizability is constrained by the homogeneous cohort (e.g., breast cancer patients). Future trials should include diverse populations and compare multiple aromas to identify optimal candidates.

It is important to note that this study was conducted exclusively on women with breast cancer referred to a specific clinic (Motahari Clinic); thus, caution should be exercised when generalizing the results to other populations, including men, patients with different types of cancer, or those receiving care in diverse healthcare settings. Additionally, cultural and individual differences in responses to aromatherapy may influence the applicability of these findings.

These results align with existing evidence suggesting that aromatherapy can play a role in mitigating chemotherapy-related side effects. Nevertheless, it is crucial to emphasize that aromatherapy was used as an adjunct to standard antiemetic medications and should not replace pharmacological treatments. Furthermore, while this approach is generally safe and cost-effective, it may not be suitable for individuals with olfactory sensitivities or allergies to citrus-based products.

### Implications for clinical practice

This study advances the body of literature pertaining to possible benefits of using aromatherapy with Citrus aurantium as an adjunct therapy for managing chemotherapy-induced nausea and vomiting (CINV) in a contemporary multicultural model of early intervention with conventional treatment. The findings are also relevant, particularly with a clinical practice perspective, in terms of improving the multidisciplinary framework within cancer treatment.

The results reinforced use of aromatherapy with Citrus aurantium in the “delayed phase” (3–5 days) after chemotherapy, this drew attention to the note reported in the CINV literature regarding CINV’s reasons for specifically aromatherapy as adjunctive therapy in symptom management, the also concurrent effect of C. aurantium to potentially displace some nausea and vomiting, absent known concurrent evidence could reflect a more cost-effective prescriptive subtractive influence in opposite to conventional medicated sedative(s) labelled as cost- effective, additionally increased potential to facilitate care collaboration from the patient correspondingly with care by their healthcare provider.

Even though this method of treatment didn’t give the patients any more negative experiences than the mainstream medicines they were suffering from when they received relief from their symptoms and/or side effects, it may have been more comfortably incorporated into the current guidelines, protocols of treatment. Second, these finder rights validate the belief about embracing the idea of personalizing care within oncology departments. If health care providers take the patient and, in turn, the cultural components into consideration, there is so much variability in creativity that could alter the patient’s experience and/or compliance. For example, patients who did not comply with antiemetic pharmacotherapy due to ineffectiveness or the side effect of the medication may find the option of aromatherapy or any means therapy part of a symptomatic control strategy for relief. Regardless of this formal structure, if you think about a structured evidence-based clinical decision-making format that could influence the development of formalized guidelines or algorithms on the use of Citrus Aurantium aromatherapy, it would hopefully complement any other various therapies. The guidelines on aromatherapy with Citrus Aurantium, would provide guidance not only to clinicians but also to the multidisciplinary team to make standardized, but customizable decision making around CINV. One could also evolve the guidelines and visual algorithm as continuing education piece for healthcare practitioners about employ aromatherapy in conjunction agricultural means.

Although this research study is specifically focused on individuals with breast cancer in AC-T chemotherapy, the results should encourage research in other populations, and cancers, to expand the research agenda, allowing for adjunct therapies to be included to a holistic approach to cancer care. Future studies should include effect size measures as well as p-values because it becomes especially useful when considering the clinical utility of adjunct therapies (such as aromatherapy) if the minimal clinically important change is not known and lack of inclusion of effect size measures hinders the analysis.

### Limitations

Several limitations should be considered when interpreting the findings. First, the absence of pre-intervention CINV assessments may have influenced the accuracy of symptom reporting. Second, a critically significant limitation is the lack of baseline Rhodes Index score. While all participants had undergone some form of chemotherapy-induced nausea and/or vomiting, prior to study entry which enabled eligibility by demonstrating they had experienced these symptoms; there was no standardized Rhodes assessment prior to the intervention. This was based on the study being outpatient in design, and allowed to manage the logistics. As a result, analyses of symptom change within groups needed to be interpreted with caution relative to the temporal nature of intra-group symptoms change analyses.

A further limitation of this study is the lack of a defined minimal clinically important difference (MCID) for the Rhodes Index, creating a barrier to clinical interpretation of the symptom changes. Future research could derive a cancer-specific MCIDs for the Rhodes Index by utilizing patient-reported outcomes and anchor-based methods.

## Conclusion

Aromatherapy with C. aurantium significantly reduced the mean severity of nausea, vomiting, and retching during the delayed phase of chemotherapy in the intervention group compared with the control group. While symptom intensity varied among individuals in both groups—likely due to factors such as overall health, treatment response, psychosocial influences, and environmental changes—the observed reduction in the intervention group suggested a beneficial effect of aromatherapy. Given its accessibility, safety profile, and cost-effectiveness, C. aurantium aromatherapy may be considered a valuable complementary therapy for managing CINV in BC patients. In light of the convenience of Citrus aurantium peer review indicates Citrus aurantium aromatherapy might be a complementary modality for helping with CINV, especially in the delayed CINV phase (i.e., days 3–5). Considering reduced symptom scores for vomiting and total Rhodes Index scores having a large effect sizes, provided rationale for the real-world value of Citrus aurantium from a therapeutic context. The findings also suggest that using one category of intervention (Citrus aurantium) does not stop at statistical significance, in other words, clinicians should consider all clinically relevant effect change.

However, further clinical trials with larger sample sizes and more complex designs are needed to confirm these findings and evaluate the long-term efficacy of this intervention.

## Supplementary Information

Supplementary Material 1.

## Data Availability

Data is provided within the manuscript or supplementary information files.

## Abbreviations

BC: Breast cancer
CAM: Complementary and alternative medicine
CINV: Chemotherapy-induced nausea and vomiting
HEC: Highly emetogenic chemotherapy
